# Intraspecific variation of residual heterozygosity and its utility for quantitative genetic studies in maize

**DOI:** 10.1186/s12870-018-1287-4

**Published:** 2018-04-19

**Authors:** Nannan Liu, Jianxiao Liu, Wenqiang Li, Qingchun Pan, Jie Liu, Xiaohong Yang, Jianbing Yan, Yingjie Xiao

**Affiliations:** 1 National Key Laboratory of Crop Genetic Improvement, Huazhong Agricultural University, Wuhan, 430070 China; 2 College of Informatics, Huazhong Agricultural University, Wuhan, 430070 China; 3 National Maize Improvement Center of China, Beijing Key Laboratory of Crop Genetic Improvement, China Agricultural University, Beijing, 100193 China

**Keywords:** Residual heterozygosity, RH hotspots, Genetic factors, HIF library for QTL fine mapping, *Zea may*

## Abstract

**Background:**

Residual heterozygosity (RH) in advanced inbred lines of plants benefits quantitative trait locus (QTL) mapping studies. However, knowledge of factors affecting the genome-wide distribution of RH remains limited.

**Results:**

A set of 2196 heterogeneous inbred family (HIF) maize lines derived from 12 recombinant inbred line (RIL) populations was genotyped using the Maize50K SNP chip. A total of 18,615 unique RH intervals were identified, ranging from 505 to 2095 intervals per population, with average maize genome coverage of 94.8%. Across all populations, there were 8.6 RH intervals per HIF line on average, ranging from 1.8 to 14 intervals; the average size of an RH interval was approximately 58.7 Mb, ranging from 7.2 to 74.1 Mb. A given RH region was present in an average of 5 different individuals within a population. Seven RH hotspots, where RH segments were enriched in the genome, were found to be subject to selection during population development. The RH patterns varied significantly across populations, presumably reflecting differences in the genetic background of each population, and 8 QTLs were found to affect heterozygosity levels in the RH hotspots. The potential use of this HIF library for the fine mapping of QTLs was assessed based on publicly available QTL information, achieving a ≤ 1 Mb resolution on average.

**Conclusion:**

The examined library of HIF lines offers insight into the RH landscape and its intraspecific variation and provides a useful resource for the QTL cloning of important agronomic traits in maize.

**Electronic supplementary material:**

The online version of this article (10.1186/s12870-018-1287-4) contains supplementary material, which is available to authorized users.

## Background

Maize (*Zea mays* L.) is one of the most important staple crops employed in food, livestock feed, and biofuel production worldwide. Maize grain production has increased more than eightfold since the beginning of the twentieth century, largely due to hybrid breeding [1]. F_1_ hybrids exhibit improvements in fitness and robustness relative to inbred lines [2]; this phenomenon is known as hybrid vigor or heterosis. Several hypotheses have been proposed to explain the underlying genetic mechanisms of heterosis. The dominance hypothesis states that slightly recessive detrimental alleles are masked by dominant alleles, resulting in superiority of a hybrid over its parents; under the over-dominance hypothesis, the synergistic effects of two alleles at individual sites result in superiority over the parents; and pseudo-over-dominance results from repulsion-phase linkage of two dominant alleles and epistasis effects between two or more loci [3, 4]. In theory, the heterozygosity of a self-crossed progeny is reduced by one-half per cycle of inbreeding, decreasing to a low level after 5 generations of inbreeding (~ 3%). The heterozygosity observed in advanced inbred progeny, known as residual heterozygosity (RH), does not comply with the law of Mendelian segregation, in that excessive RH is observed in some genomic regions. In a maize nested association mapping (NAM) population including a set of 25 recombinant inbred line (RIL) populations that had undergone more than 5 cycles of self-crossing, a higher level of heterozygosity was observed in pericentromeric regions across all populations and chromosomes relative to telomeric regions, possibly resulting from the selective preservation of heterozygosity due to the pseudo-over-dominance of heterosis for yield QTLs in recombination-inhibited regions [5, 6]. In a set of European maize lines and their American counterparts, deleterious mutations were reported to be enriched within heterozygous segments in comparison with the rest of the genome, and selection therefore helped maintain RH against inbreeding depression [7]. Similar results obtained across different germplasms imply that RH may benefit organisms during natural or artificial selection. However, knowledge of the factors affecting the genome-wide distribution and biological implications of RH remains limited. The identification of genes underlying traits of agricultural and economic importance is a long-term objective that will enhance the understanding of the architecture of complex traits and accelerate crop genetic improvement. To this end, two methods have been routinely used in plants. In model species, the gene-driven reverse genetics approach, based on the availability of large mutagenized libraries, has been employed to explore phenotypes associated with a specific mutated gene variant. In maize, tools such as transposons, T-DNA, ethyl methane sulfonate (EMS) mutagenesis, and genome editing have been commonly used to develop mutant libraries [8]. However, mutagenesis usually results in loss-of-function mutations, causing a dramatic phenotypic change that is often deleterious and cannot be directly applied in breeding programs. Map-based cloning, a phenotype-driven forward genetics strategy, has been proven to be an effective approach for isolating genes for qualitative and quantitative traits in model plants and important economic crops [9]. For QTL fine mapping (i.e., ≤200 Kb), it is routine to use a large segregating population of near-isogenic lines (NILs), at the cost of advanced backcrossing efforts [10, 11]. Alternatively, RH in an RIL population provides an attractive opportunity to narrow QTL regions without extensive backcrossing, which is referred to as the heterogeneous inbred family (HIF) approach [12]. The HIF approach takes advantages of the recombination events within a region of RH in an RIL population (comprising ca. ~ 3% of the total genome in the F_6_ generations) to narrow a QTL interval, thus avoiding background noise if only one target QTL is located in the RH region. Thus far, the HIF approach has been successfully applied to QTL cloning for many important traits related to plant morphology, diseases, flowering time, and seed weight in several species [13–15].

In the present study, a set of twelve advanced inbred populations comprising more than 2000 lines was used, including eleven RIL populations (≥F_6_) and one BC_2_F_5_ population [16]. All materials were genotyped using the commercial MaizeSNP50 chip [17]. These data make it possible to capture the genome-wide landscape of RH across diverse genetic backgrounds and to study the biological relevance of RH hotspots for agronomic traits. Furthermore, we demonstrated the robust potential of a diverse HIF library for the fine mapping and cloning of QTLs controlling important agricultural traits in maize.

## Methods

### The twelve linkage populations and genetic linkage maps

A total of 12 advanced inbred populations were employed to explore the patterns of RH across the whole genome, including one F_10_ RIL (ZONG3/YU87–1), one F_9_ RIL (B73/BY804), nine F_6_ RILs (BY815/KUI3, DAN340/K22, DE3/BY815, K22/CI7, K22/BY815, KUI3/B77, KUI3/SC55, YU87–1/BK, and ZHENG58/SK), and one BC_2_F_5_ population (MO17/X26–4). The pedigree and origins of these 17 parent lines have been described previously [16]. For each line in a population, two well-developed plants with morphologies similar to their siblings were selected, and their leaves were collected separately. The plant that bore more seeds was genotyped using the Illumina MaizeSNP50 BeadChip, containing 56,110 markers derived from the B73 reference sequence [18]. The seeds from each genotyped plant were maintained separately for future development of heterogeneous inbred families. Other seeds from sibling plants were stored in bulk for later genetic analysis. The construction of the twelve ultra-high-density linkage maps has been described previously [16]. To facilitate the genome-wide evaluation of RH, we corrected regions where the physical positions of SNPs were not collinear with their genetic positions via the linear interpolation method, taking into account the physical positions of flanking collinear SNPs. The obtained genetic maps captured the majority of recombination events, and the missing marker genotypes were imputed using the closest flanking non-missing markers. The details of these procedures have been described previously [19]. The genotypic data for all 12 populations are publicly available at a permanent website (http://www.maizego.org/Resources.html).

### Evaluation of RH across the genome and between populations

Using the raw genotypes, each SNP marker could be classified as homozygous or heterozygous. In each line, the physical region spanned by continuously heterozygous markers was defined as the RH interval. The boundary of each RH interval was estimated as halfway between the homozygous and heterozygous markers nearest the recombinant point. To avoid statistical bias due to pollen contamination, the whole set of 2319 lines was evaluated, and lines exhibiting an extreme excess of heterozygosity were removed based on the empirical distribution of the residual heterozygosity rate (RHR) and the carrying RH length of the lines. In detail, lines with the following characteristics were excluded from further analysis: 1) RHR exceeding 10% (mean plus 1.6 times SD; Additional file 1: Figure S1a), or 2) RHR exceeding 9% (mean plus 1.3 times SD; Additional file 1: Figure S1a) and any single RH interval spanning more than 50 Mb (Additional file 1: Figure S1b). Overall, a total of 2162 inbred lines (139–196 per population) were retained for analysis (Table 1). To calibrate statistical bias in the case of genotyping mistakes, we revised the typed homozygous intervals between two RH intervals only if they covered ≤3 homozygous markers and spanned ≤500 Kb, or vice versa, given that the average length between two recombination breakpoints was more than 500 Kb [16].

**Table 1:** Summary of RH intervals in the heterogeneous inbred family library

Population	Pedigree	Lines^a^	RHN^b^	RHN/line^c^	RH (Mb)^d^	RHR (%)^e^
KUI3/SC55	F_6_	168	1922	11 (4–44)	6.3 (0.03–111.7)	3.52 (0.35–9.56)
YU87–1/BK	F_6_	139	1969	14 (3–62)	5.2 (0.04–78.8)	3.57 (0.23–9.25)
DE3/BY815	F_6_	190	1814	9 (2–21)	7.8 (0.12–81.1)	3.6 (0.07–9.44)
K22/BY815	F_6_	193	1413	9 (1–21)	7.4 (0.04–97.8)	3.39 (0.12–9.45)
BY815/KUI3	F_6_	184	2095	11 (1–27)	6.6 (0.05–89.7)	3.6 (0.19–9.95)
K22/CI7	F_6_	177	1507	8.5 (0–57)	6.8 (0.03–114.1)	2.8 (0–8.25)
DAN340/K22	F_6_	183	1672	8.5 (0–44)	6.9 (0.02–152.9)	3.07 (0–9.10)
KUI3/B77	F_6_	166	1873	11 (2–53)	6.6 (0.02–91.9)	3.59 (0.06–9.86)
ZHENG58/SK	F_6_	196	1843	9 (1–25)	6.3 (0.02–70.0)	2.89 (0.07–9.58)
B73/BY804	>F_8_	190	1143	6 (0–49)	5.4 (0.004–91.0)	1.61 (0.01–7.51)
ZONG3/YU87–1	>F_8_	193	505	1.8 (0–27)	2.7 (0.01–45.6)	0.35 (0–7.7)
MO17/X26–4	BC_2_F_5_	183	859	4.7 (0–18)	9.7 (0.05–172.2)	2.20 (0–8.89)

^a^Number of lines in each population

^b^Number of residual heterozygosity intervals in each population

^c^Average and range of the number of residual heterozygosity intervals per line in the population

^d^Average and range of the length of residual heterozygosity intervals in each population

^e^Average and range of residual heterozygosity rate per line in each population

The global distribution of RH was evaluated using a 5 Mb window with 1 Mb walking steps in each population. In each 5 Mb window, the RHR was calculated as the summed length of RH intervals divided by the window size and the number of lines for each population; the number of RH intervals (RHNs) was calculated as the count of RH intervals divided by the window size. Additionally, we estimated the recombination rate (RR) as the genetic distance divided by the physical distance in each window (cM/Mb). Furthermore, we compared heterozygosity levels between the pericentromeres and the remaining chromosome arms, where the region spanning 10 cM upstream and downstream of the centromere was defined as the pericentromere [6]. It must be noted that we defined the heterozygosity level as the proportion of heterozygous markers divided by all tested markers, to compare the results with those for the NAM population [6]. To explore whether marker density affects the level of heterozygosity, we examined two levels of marker density. One included all of the markers in the genetic maps, and the other included 1100 markers that were randomly selected from the whole set with three replications. In the set of 1100 random markers, 350 and 750 markers were separately selected in the pericentromeres and remaining chromosome arms, respectively, comparable to the NAM population [6].

### QTL analysis of RH hotspots across populations

As RHR varied widely across the whole genome in each population, we performed a permutation analysis to identify statistically significant RH hotspots across the genome. The threshold of RHR across the genome was established via permutation as follows. In each permutation, we first randomly selected one half of the lines and blocks of markers in sliding windows of 5 Mb were simultaneously shuffled for these lines, while maintaining the original markers in the other half of the lines, to obtain a randomly distributed genotype dataset. In addition, the average RHR for all of the lines in each window was calculated and then ordered from largest to smallest. Unlike the conventional permutation procedure, we recorded the second largest (i.e., 99th maximal) RHR score among the 5 Mb windows, rather than the maximum RHR score, to reduce the possible impact of outliers [20]. This procedure was repeated 1000 times, and a null distribution of the empirically recorded RHR values based on 1000 permutations was obtained. It is worth noting that when shuffling the blocks of markers in all lines at once, the recorded RHR values across 1000 permutations will consistently be the same, which is why we chose the above-described method. At a genome-wide error rate of 0.01, a threshold of RHR ranging from 2.05% (ZONG3/YU87–1) to 10.58% (K22/BY815) was declared for the existence of an RH hotspot in a given population. For simplicity, the physical range delimited by the RHR threshold was defined as the confidence support region of an RH hotspot.

The maize genome consists of abundant duplicated sequences, such as repetitive sequences and gene paralogs [18], which probably increase heterozygous genotype calling. To test the possibility of the presence of RH artifacts due to SNP calling, we performed an enrichment analysis by comparing the frequency of duplicated sequences within each RH hotspot with the expected values obtained by dividing the size of each RH hotspot by the maize genome size, assuming that duplicated sequences were randomly distributed across the genome. The binomial distribution was employed to test the null hypothesis that the observed proportion of duplicated sequences within each RH hotspot was greater than the proportion expected by chance (*P* < 0.05; right-tailed). For each RH hotspot, to precisely evaluate the potential bias, we analyzed the enrichment of repetitive sequences and gene paralogs separately after filtering the untagged duplicated sequences by polymorphic markers in specific populations. In addition to the analysis of all tagged paralogs, we also filtered out paralogs showing a similarity less than 80%, to reduce ascertainment bias in non-B73 inbred lines.

To explore whether random evolutionary forces such as genetic drift could produce the observed RH pattern in the genome without selection, we performed a simulation analysis in which we attempted to obtain an expected RH pattern by chance, driven by recombination and drift. We ignored mutations in this analysis, given the short period of development for the RIL populations. We simulated the genotypes successively to mimic the consequences of drift and recombination in the K22/BY815 population, including 400 lines during 5 generations of self-pollination. The simulation procedure was as follows:

1. We assume that the inherent probability that crossover realistically occurs in one interval during one meiosis is stable for all generations, which is designed as the expected recombination frequency (*f*_e_) for one interval;
2. In reality, a given crossover is not always detectable, since only the crossovers taking place within heterozygous intervals can be detected. Thus, the probability of detecting a given crossover (*f*_g_) decreases 50% per selfing generation, following a geometrical sequence with an initial value of *f*_*e*_ and a common ratio of 1/2. The *g*-th term of the sequence is given by *g* is the index of the generation;
3. The observed recombination frequency (*F*_*obs*_) in the F_6_ generation for a given interval should correspond to all detectable recombination events during gamete meiosis across the previous generations (F_1_-F_5_). As a result, *f*_e_ is calculated as one-half of *F*_*obs*_, while *F*_*obs*_ can be estimated via the genetic map in the F_6_ generation;
4. In one meiosis for the *g*-th generation, a crossover that realistically occurs within a given interval in one gamete could theoretically be considered a random event that follows a binomial distribution, B(*n*, *f*_e_), where *n* is the number of gametes, and *f*_e_ is the expected recombination frequency. To simulate the haplotype of female and male gametes, we ignored the situation of crossover interference and independently drew the recombination positions per interval on the specific binomial distribution. We then randomly fused female and male gametes to generate diploid genotypes at the (*g* + 1)-th generation;
5. Following the SSD procedure, we initiated the simulation from the F_1_ gametes and the derived F_2_ diploids, and we successively repeated this simulation for 5 generations to obtain (*g* + 1)-th diploids from *g*-th gametes up to F_6_ diploids.
6. To assess the reliability of the simulation analysis, we randomly selected 193 lines, equivalent to the real population size, from 400 lines to avoid the influence of genetic drift, and we recorded the average heterozygosity level and the observed recombination per line for each simulated generation from F_2_ to F_6_; additionally, for the F_6_ progenies, the relative RHR was calculated as the simulated RHR in the hotspot regions (Hot2 and Hot3) minus the average RHR in the whole genome;
7. This procedure (step 4―6) was repeated 100 times, producing the simulated distribution of heterozygosity, the mean recombination per line for each generation and the null distributions of the relative RHR at hotspots. The *t*-test was used to evaluate whether the relative RHR observed for the two hotspots was derived from the null distribution (*P* < 0.05).

To explore the genetic factors underlying RH variation, we performed QTL analysis of RHR in RH hotspot regions. In each hotspot, the RHR for each line was treated as a phenotype for QTL analysis. The composite interval mapping procedure [21] implemented in Windows QTL Cartographer v.2.5 software was employed [22]. We chose an LOD score of 3 as the threshold to declare the significance of a QTL. The confidence interval of a QTL was defined as a two-LOD drop region from the QTL peak. A QTL was defined as a cis-hQTL if it overlapped with its RH hotspot and was otherwise defined as a trans-hQTL.

### Assessment of the value of RH for agronomic QTLs identified in 10 populations

To assess the value of the HIF library for quantitative trait analyses based on empirical data, we collected 1191 QTLs for 19 agronomic traits that had been previously identified in all populations except for KUI3/SC55 and MO17/X26–4, for which no QTL data were available (Additional file 2: Data S1) [19]. For each QTL, we calculated three statistics to demonstrate the potential of the HIF library to narrow the QTL region: coverage, resolution, and depth. The coverage for a QTL is the average proportion of all QTL intervals that is jointly covered by heterozygous intervals in one population. The resolution for a QTL defines the average interval sizes for all QTLs delimited by the different available RH intervals. The depth of a QTL indicates the average number of RH intervals that cover or partially cover all QTLs.

## Results

### The genome-wide landscape of intraspecific variation in maize RH lines

A total of 12 advanced inbred populations were used to explore the patterns of RH across the whole genome. The procedures employed for the genotyping and construction of a genetic linkage map for each population have been described previously [16] and ultimately covered 11,360 to 15,285 polymorphic markers across all of these populations. Detailed information for each population is provided in Table 1. A total of 18,615 RH intervals were detected in all lines from the 12 populations, with an average of 1551 intervals per population, ranging from 505 to 2095 (Table 1). The RH intervals for each line within the 12 populations spanned approximately 58 Mb in length on average, or 2.8% of the B73 reference genome [18], and for each line, the ratio of heterozygous interval length to genome length ranged from 0 to 9.95% in the F_6_ populations and from 0 to 8.89% in the RIL and backcross populations. In the nine F_6_ populations, the observed RH of 3.34% (i.e., the average heterozygosity of all of the lines in nine populations) was significantly higher than the expected value (3.125%; ANOVA, *P* < 0.001), whereas there was no significant inflation of RH beyond expectations for the remaining three populations (Table 1). On average, 10 RH intervals per line were observed in the F_6_ populations and 4 in the RIL and backcross populations, with average lengths of 6.7 and 4.1 Mb per interval, respectively (Table 1).

**Fig. 1:**
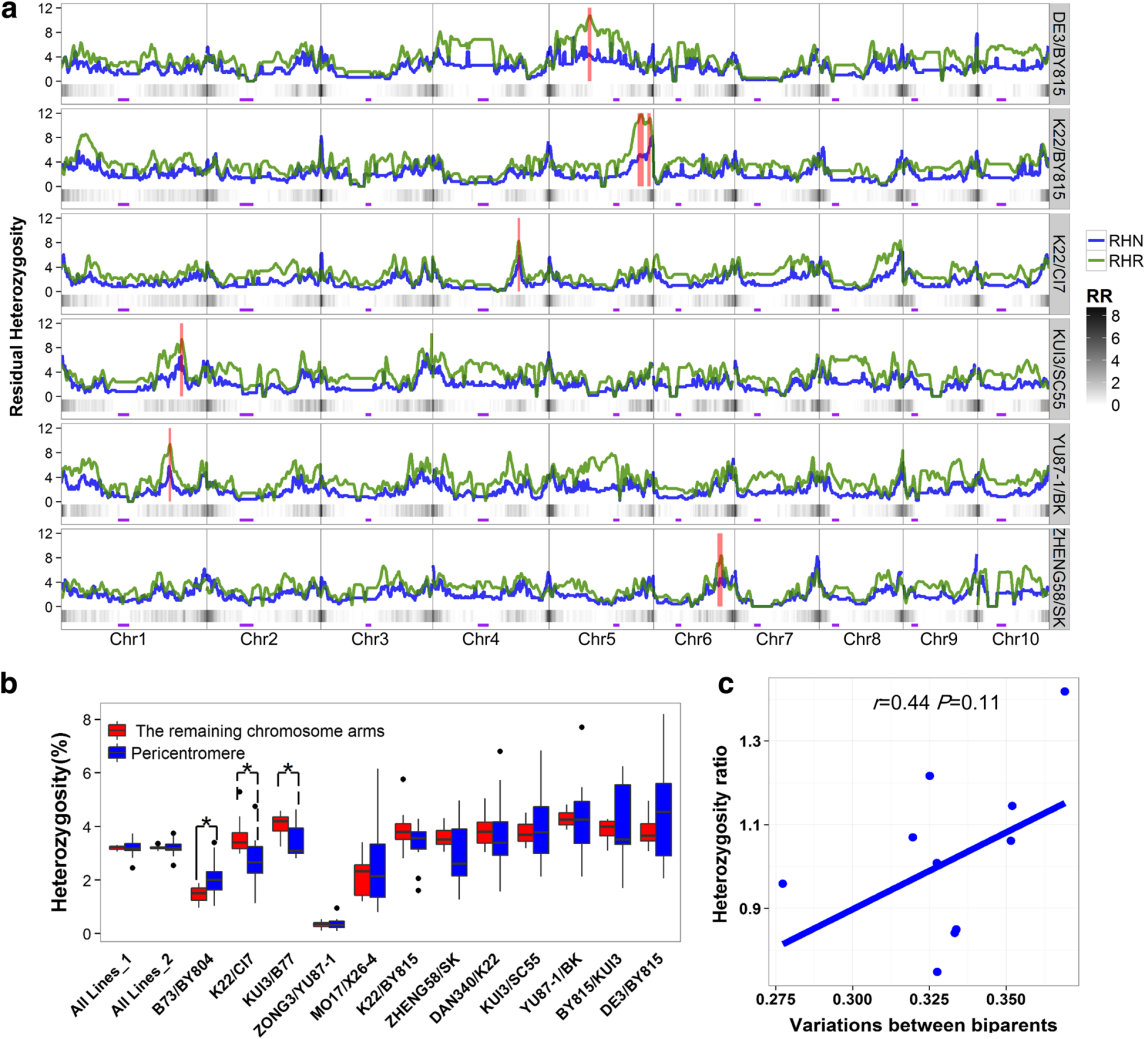
The genome-wide landscape and intraspecific variations of RH in maize. (**a**) Genome-wide distribution of RH across populations. The 6 populations in which RH hotspots were identified are illustrated, and the 7 RH hotspots are highlighted with red bars. The blue lines indicate the number of RH intervals (RHN), and the green lines indicate the RH rate (RHR). The purple rectangles indicate the approximate position of the centromere for each chromosome, and the recombination rate (RR) is shown as a heatmap bar below each population. (**b**) Comparisons of heterozygosity levels between the pericentromere and the remaining chromosome arms across 12 populations. “All Lines_1” indicates that all polymorphic markers were used to perform the comparison by joining all lines in 12 populations, while “All Lines_2” indicates that only the randomly selected 1100 markers from “All Lines_1” were employed to evaluate the impact of marker density on the comparison. The populations marked with a solid line and asterisk show a significantly higher heterozygosity level in the pericentromere than in the remaining chromosome arms, while the populations marked with a dotted line and asterisk show a significantly lower level of heterozygosity in the pericentromere than in the remaining chromosome arms. (**c**) The correlation between the heterozygosity ratio and the proportion of polymorphic markers in the two parents for 12 populations. The heterozygosity ratio is defined as the level of heterozygosity in the pericentromere divided by that in the remaining chromosome arms

To evaluate the variation of RH across the whole genome, we employed two statistical parameters to measure the level of RH within a 5 Mb sliding window: RHR and RHNs (details in Methods). For each population, RHR and RHNs were found to be unevenly distributed between and along chromosomes (Fig. 1a and Additional file 3: Figure S2). The trends between RHR and RHN were similar, with Pearson correlations ranging from 0.57 to 0.79 across populations (*P* < 0.001). We identified 7 unique RH hotspots located on chromosomes 1, 4, 5 and 6 in the DE3/BY815, K22/BY815, K22/CI7, KUI3/SC55, YU87–1/BK, and ZHENG58/SK populations that showed significant excess heterozygosity relative to what would be expected by chance (permutation test, *P* < 0.001; Fig. 1a and Table 2). RHR for these 7 regions varied between 8.0% and 11.3%, which was approximately three times higher than the average value for the population (Table 2). Considering the complexity of the maize genome, to examine possible artifacts of RH due to SNP calling, we performed an enrichment analysis by comparing the frequency of duplicated sequences (including paralogs and repetitive sequences) within each RH hotspot with the values expected by chance. We found that none of the RH hotspots were significantly enriched in paralogous genes (*P* > 0.5) according to the critical criteria, although Hot7 was an exception (*P* = 9.6 × 10^− 3^) due to a loose criteria (Additional file 4: Figure S3a and b). Similarly, repetitive sequences were not enriched in all hotspots (*P* = 1; Additional file 4: Figure S3c). This result implied that the identified RH hotspots were not likely to be caused by genotype artifacts based on duplicated sequences.

**Table 2:** Hotspots of residual heterozygosity and hQTL across populations

	RH hotspot				hQTL					Traits^e^	
	Population^a^	Location (Mb)	RHR (%)^b^		Interval (Mb)^c^	LOD	*R*^2^ (%)	Additive^d^	QTL type	RH hotspot^f^	hQTL
Hot1	DE3/BY815	Chr5:81–89	10.6/3.7	hQTL1	Chr5:68.8–92.4	7.2	14.5	0.182^♀^	cis	–	–
				hQTL2	Chr5:151.7–159.3	14.3	28.2	0.213^♀^	trans	–	–
Hot2	K22/BY815	Chr5:182–197	11.3/3.4	hQTL3	Chr5:204.2–208.1	8.2	17.7	0.122^♀^	trans	TBN, HKW	TBN
Hot3	K22/BY815	Chr5:204–211	11.0/3.4	hQTL4	Chr5:208.1–208.9	13.3	19.0	0.094^♀^	cis	ULA, HKW, KW, KT	KW
Hot4	K22/CI7	Chr4:174–181	8.0/2.8	hQTL5	Chr5:22.1–29.3	4.4	9.2	0.080^♂^	trans	LNAE	–
Hot5	KUI3/SC55	Chr1:246–251	9.4/3.5	hQTL6	Chr1:243.8–247.2	4.3	8.3	0.072^♂^	cis	na	na
Hot6	YU87–1/BK	Chr1:221–227	9.3/3.6	hQTL7	Chr1:260.9–269.2	6.1	15.6	0.134^♂^	trans	LW	–
Hot7	ZHENG58/SK	Chr6:136–144	8.2/2.9	hQTL8	Chr3:224.0–224.5	3.4	6.4	0.062^♀^	trans	KNPR, EL, EW	–

^a^Population in which the specific RH hotspot was identified; the name of the population is the names of the female and male parents separated by a backslash

^b^Average residual heterozygosity rate within the hotspot region (before backslash) and at the genomic level (after backslash) for each population

^c^Chromosome and physical location of the hQTL for each hotspot

^d^Additive effect of each hQTL; a value marked with “♀” indicates that the allele from the female parent increases RHR, while a value marked with “♂” indicates that the allele from the male parent increases RHR in the hotspot

^e^The trait represents the collected phenotypes that exhibited significant differences either between heterozygotes and homozygotes within RH hotspots or between two homozygous genotypes for hQTLs (*P* < 0.05). “-” indicates that no significant trait existed for this hotspot; and “na” indicates that no phenotypic data were available in this population. TBN: tassel branch number. HKW: one hundred kernel weight. ULA: upper ear leaf angle. KW: kernel width. KT: kernel thickness. LNAE: leaf number above ear. LW: leaf width. KNPR: kernel number per row of ear. EL: ear length. EW: ear weight

^f^For underlined traits, the heterozygous genotypes show higher phenotype values than the recessive homozygous genotypes (*P* < 0.05) but present no difference from the dominant homozygous genotypes, while for traits that are not underlined, the heterozygous genotypes contribute significantly to higher phenotype values than the genotypes of both homozygotes (*P* < 0.05). In this analysis, the dominant and recessive homozygotes were defined as the homozygotes responsible for the higher and lower phenotype values per trait, respectively

To further explain the observed patterns of RH, we employed simulation analysis to assess whether random evolutionary forces, such as drift, could produce the observed patterns without selection. Starting from F_1_ gametes, we simulated the diploid genotypes successively to mimic the consequences of drift and recombination in the K22/BY815 population through 5 selfing generations. We plotted the distribution of average heterozygosity and recombination events per line at the genome-wide level for each generation based on 100 simulations and found that the average heterozygosity decreased by approximately 50% per selfing generation and up to ~ 3% in F_6_ (Additional file 5: Figure S4a), which was very close to the expected value (3.25%) in theory. In addition, the mean recombination per line gradually increased across generations, and the increment between continuous generations followed a geometrical sequence with the common ratio of 1/2, ultimately producing 34 recombination events per line in the F_6_ genome (Additional file 5: Figure S4b), which was within the limit of real recombination events (29―49) (Additional file 5: Figure S4b) [16]. These two results indicated that the simulation analysis faithfully mimicked the joint consequences of drift and recombination in the real population. In the simulated distributions, the average relative RHR for each line in the F_6_ generation was significantly lower than that captured in the real data for both RH hotspots (*P* < 2.2 × 10^− 16^; Additional file 5: Figure S4c and Additional file 5: Figure S4d), suggesting that it is impossible to obtain the observed RH pattern under random evolutionary forces (e.g., recombination and drift) unless selection is involved in this process.

Similar to previous reports [16], we found that recombination fluctuated significantly across the chromosomes (Fig. 1a), which led us to test the relationship between the recombination rate (RR) and genome-wide RH levels (RHR and RHN) in each population. Interestingly, we consistently found significantly positive correlations between RR and RHN in all twelve populations (Additional file 6: Figure S5). These correlations indicated that recombination may act as one factor affecting the genome-wide RH pattern, implying that RH regions that were smaller and occurred in greater numbers were present in high-recombination regions, whereas larger and fewer RH regions occurred in low-recombination or centromeric regions. However, RR showed significant negative correlations with RHR in two populations, positive correlations in eight populations, and no significant correlation in two populations (Additional file 6: Figure S5). This diverse pattern of RHR across populations suggests that factors other than RR may affect the variation of RHR; thus, RHR was used in further analyses to further explore RH. More precisely, the pericentromeres and remaining chromosome arms were compared in terms of RHR. Notably, we found no significant difference in RHR between pericentromeres and the remaining chromosome arms when all of the lines in the 12 populations were examined together (Fig. 1b). This pattern was inconsistent with previous findings in two NIL populations and the NAM population consisting of 25 RIL populations founded by crossing 25 diverse inbred maize lines with one common parent, B73, for which a significantly higher RHR was observed in the pericentromeres than in the remaining chromosome arms [5, 6]. To improve comparability with the NAM population, we randomly selected 1100 markers from the 50 K SNPs, including 350 markers located in pericentromeres and 750 in the remaining chromosome arms. The process was repeated three times to avoid sampling bias, yet there was no significant difference in heterozygosity levels between pericentromeres and the remaining chromosome arms (Fig. 1b). Interestingly, a higher RHR was found in pericentromeres than in the remaining chromosome arms in the B73/BY804 population (2.1% vs. 1.47%; *P* < 0.05), whereas a lower RHR was observed in the K22/CI7 (2.8% vs. 3.6%; *P* < 0.05) and KUI3/B77 (3.5% vs. 4.1%; *P* < 0.05) populations, and no significant difference was detected in the other 9 populations (Fig. 1b). We also observed a large difference in the ratio of the RHR in pericentromeres to the RHR in the remaining chromosome arms in the 12 populations, even those sharing parental lines; the ratio was 1.2 for DE3/BY815, compared with 0.85 for K22/BY815. Hence, to test whether the higher RHR ratio was the result of greater sequence diversity between two founder lines, genetic differences between parents were estimated based on the 50 K SNPs. We observed nominal significance between the RHR ratios and genetic variation in the 12 populations (*r* = 0.44, *P* = 0.11; Fig. 1c), possibly due to the limited number of populations employed in the present study. Overall, the findings that RHR varied both across the whole genome and between the 12 populations implied that the distribution of RHR may be affected by genetic factors in specific populations.

### Genetic basis of RH hotspots and their relationship with agronomic traits

**Fig. 2:**
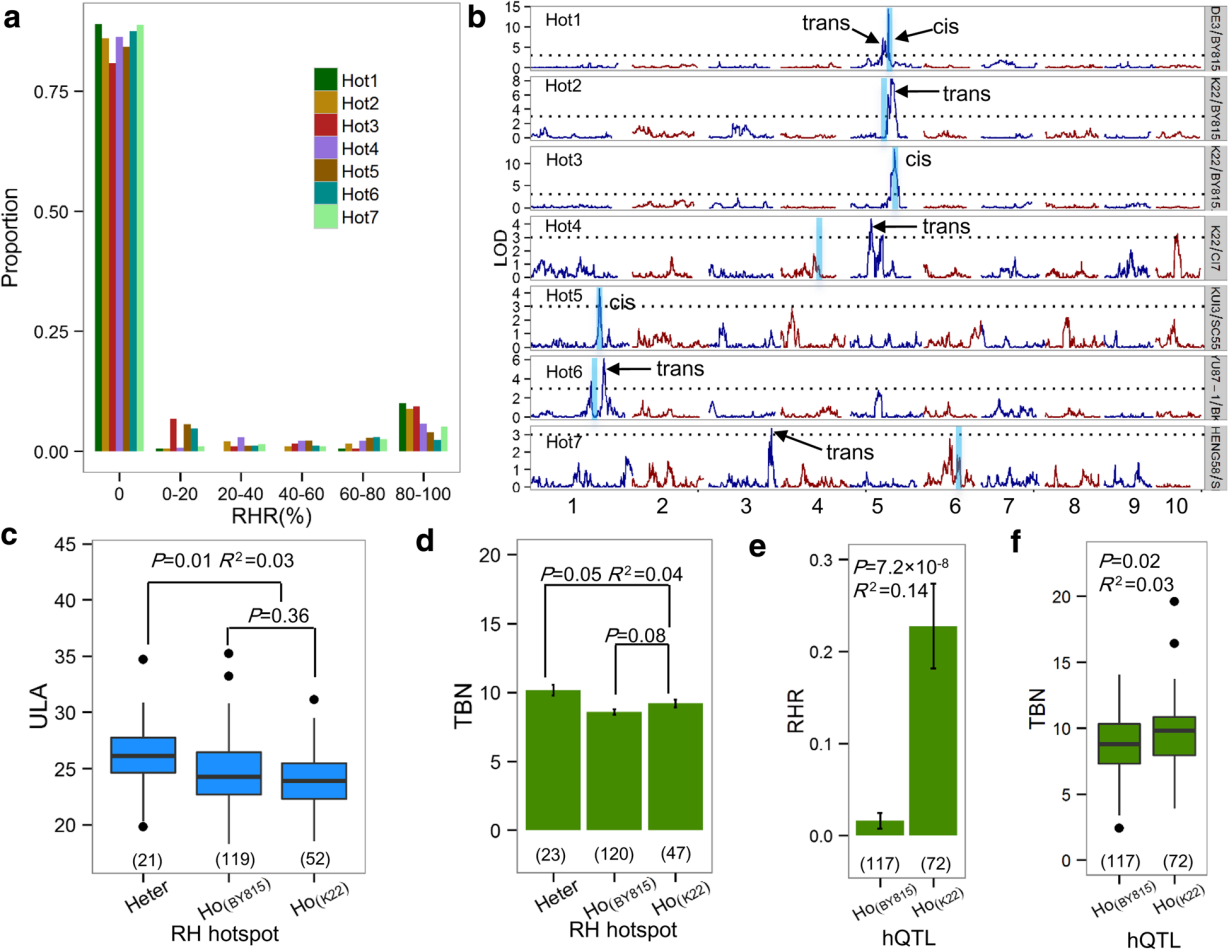
QTL analysis of RH hotspots and functional inferences for phenotypes of agronomic traits. (**a**) Distribution of heterozygosity rates within each RH hotspot. (**b**) Overview of genome-wide hQTLs for RHR in RH hotspots. Only the 6 populations with detected RH hotspots are illustrated. The blue vertical rectangles indicate the genetic position of the RH hotspots. (**c**) Phenotypic functions of the RH hotspots with a cis-hQTL. RH hots3 was coordinated by itself per se (i.e., acting as a cis-hQTL), and within the hotspot, the heterozygotes exhibited a significantly greater upper leaf angle (ULA) than any homozygote (*P* ≤ 0.01). (**d**-**f**) Phenotypic role of RH hotspots with a trans-hQTL. The heterozygotes within RH hot2 exhibited a marginally greater tassel branch number (TBN) than any homozygous type (*P* ≤ 0.05), but two homozygous types showed basically the same TBN (*P* = 0.08); data represent the mean ± standard error (se.) (**d**) A trans-hQTL regulates the 5 Mb-distant RH hot2. In this trans-hQTL, the K22 allele results in a significant increase in RHR relative to the BY815 allele at Hot2 (*P* = 7.2 × 10^− 8^); data represent the mean ± se. (**e**) In contrast, the K22 allele leads to a significantly greater tassel branch number (TBN) than the BY815 allele (*P* = 0.02) (**f**)

To further explore how genetic factors affect RH variations, we performed QTL analysis of the heterozygosity rate in RH hotspot regions (hQTL analysis). For the 7 RH hotspots, hQTL analysis was conducted in each of the 6 populations with identified RH hotspots. For the purpose of hQTL analysis, the phenotype was defined as the RHR within a specific hotspot region of each line; therefore, the phenotypic distribution was skewed due to the low overall heterozygosity (Fig. 2a). The hQTL analysis identified 8 hQTLs for all hotspots at an LOD of 3, including 3 cis-hQTLs and 5 trans-hQTLs (Fig. 2b). Among the 8 identified hQTLs, three exhibited alleles of increased effect from the male parents, while five QTLs exhibited alleles of increased effect from the female parents (Table 2), indicating that there was no preference regarding the contribution to offspring lines of hQTL alleles with high heterozygosity from male or female parents. Interestingly, the hQTLs were all specifically located in the corresponding population in which the RH hotspot was originally identified. This result directly supported the hypothesis that the distribution of RH across populations is heritable, probably reflecting the effects of these population-specific hQTLs.

Since previous reports have suggested that RH might correlate with fitness or heterosis [7], we tested this hypothesis by evaluating the phenotypic performance of heterozygotes relative to homozygotes within 7 RH hotspots for 21 agronomic traits. The information and abbreviations of the 21 traits are provided in Additional file 2: Data S1. Overall, 10 of the 21 measured agronomic traits showed significant differences between heterozygotes and homozygotes for at least one RH hotspot (Table 2). For example, in the K22/BY815 population (Figs. 1a and 2b), the heterozygotes within Hot3 (Chr5: 204–211 Mb) showed more horizontal leaves than the homozygotes (*P* = 0.01, *R*^2^ = 0.03), with an increased magnitude of the upper leaf angle (2°), but no significant differences were observed between the homozygous genotypes (*P* = 0.36; Fig. 2c); in contrast, the heterozygosity rate in Hot3 was controlled by hQTL4, a cis-hQTL (LOD = 13.3, *R*^2^ = 19%) that exactly overlaps with Hot3 (Table 2 and Fig. 2b). In addition, Hot2 (Chr5: 182–197 Mb; identified in the K22/BY815 population) heterozygotes showed a marginally significantly higher tassel branch number (TBN) than the K22 group (*P* = 0.05) and a significantly higher TBN than the BY815 group (*P* = 0.003), although TBN was similar between the two homozygous groups (*P* = 0.08) (Fig. 2d). However, the heterozygosity rate within Hot2 was not regulated by Hot2 itself but was influenced by hQTL3, a trans-hQTL (LOD = 8.2 and *R*^2^ = 17.7%) located 5 Mb away from Hot2 showing a 20-fold difference in the heterozygosity rate between the K22 and BY815 alleles (*P* = 7.2 × 10^− 8^, *R*^2^ = 0.14; Fig. 2e and Table 2), which also affected the TBN phenotype (*P* = 0.02, *R*^2^ = 0.03; Fig. 2f). Some traits, such as HKW, LNAE and LW, presented higher values in the heterozygotes at Hot2, Hot4 and Hot6, respectively, compared with lower homozygotes (*P* = 0.047, *P* = 0.0029 and *P* = 0.03) but showed no differences from other homozygotes (*P* = 0.07, *P* = 0.17 and *P* = 0.18), while the HKW, KT and KW of heterozygotes at Hot3 and the KNPR, EL and EW of heterozygotes at Hot7 exhibited higher values than those of any homozygotes (*P* < 0.05; Table 2). Among the 10 traits corresponding to different hotspots, 8 exhibited higher phenotypes in the heterozygotes than in any parent, a phenomenon known as “best-parent heterosis”, which supports the hypothesis of the association of RH with fitness and heterosis.

To explore the mechanisms underlying the relationship between RH hotspots and heterosis, we evaluated the frequencies of two alleles and recombination rates within 2 Mb on each side of the RH hotspots. Under the hypothesis of pseudo-over-dominance for explaining heterosis, the excess of heterozygotes is based on the selection of two favorable loci being linked in repulsion phase. The observation of segregation distortion of alleles towards one parent immediately before hotspots and towards the other parent immediately after the hotspots as well as fewer recombination events in heterozygous lines than in homozygous lines indicates that recombination and selection occurred to bring together the two favorable alleles in the same haplotype, which is consistent with the pseudo-over-dominance hypothesis. Our data showed that the proximal regions of three hotspot (Hot4, Hot5 and Hot7) did not carry any markers that were significantly distorted in the homozygous parents (*P* > 0.05, χ^2^ test; Additional file 7: Table S1). The proximal regions of the remaining four hotspots (Hot1, Hot2, Hot3 and Hot6) showed significantly homozygous allelic distortion (*P* < 0.05, χ^2^ test; Additional file 7: Table S1) but all markers within each region were uniformly distorted in the same parent (Additional file 8: Table S2). Accordingly, we found that the heterozygous lines tended to exhibit significantly more recombination than the homozygous lines across all hotspots (*P* < 0.01, *t*-test; Additional file 7: Table S1). These results contradict the expectations of the pseudo-over-dominance hypothesis, implying that the over-dominance hypothesis as a possible alternative contributing to RH hotspots.

### Use of the HIF library for quantitative genetic analyses

**Fig. 3:**
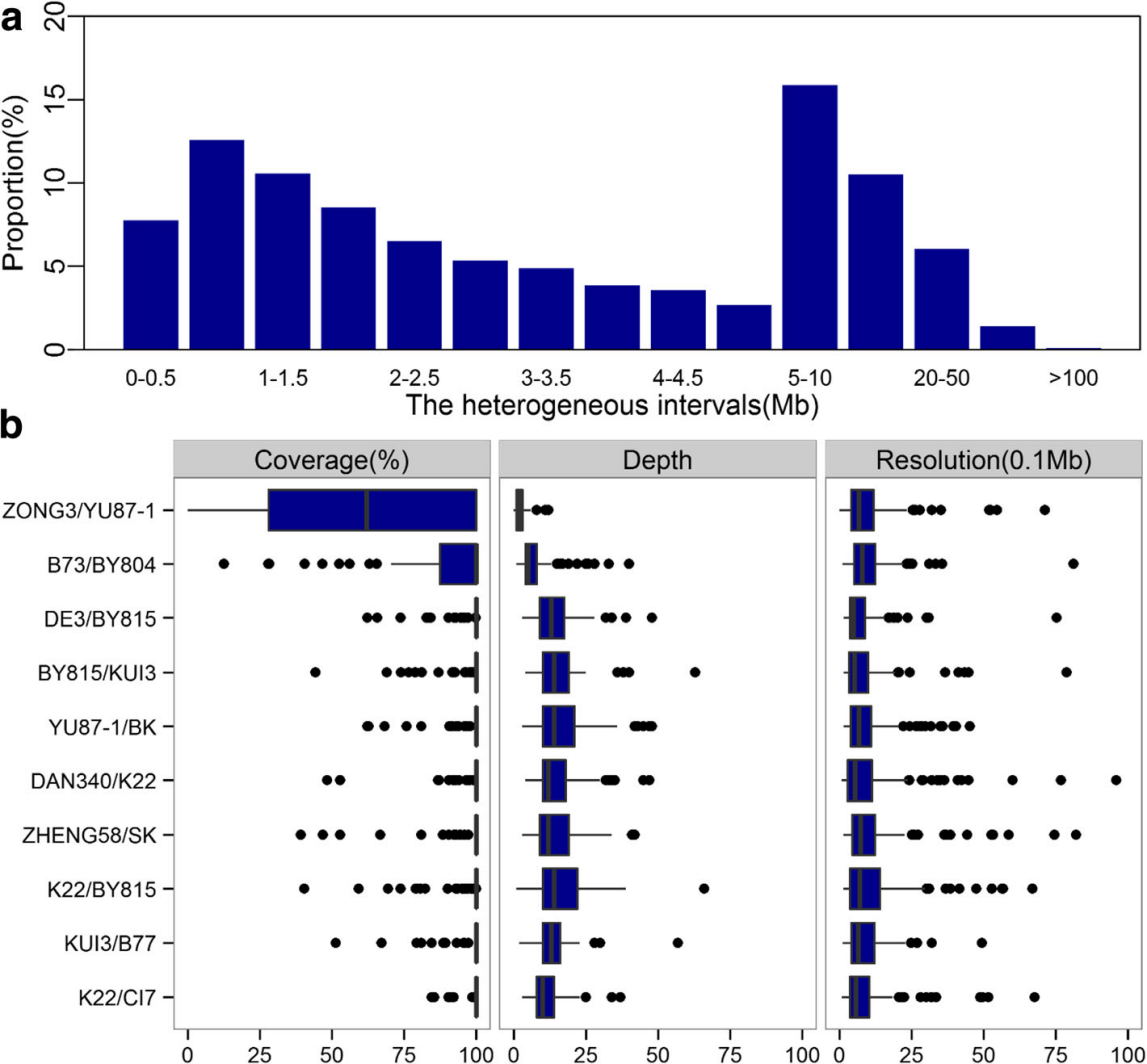
RH features of the HIF library and the empirical potential of quantitative trait studies. (**a**) Distribution of the length of RH intervals in the HIF library. (**b**) The coverage, depth, and resolution of RH for the dissection of QTLs. Ten populations were used to evaluate these parameters (no QTL information was available for the other 2 populations)

The set of 12 advanced inbred populations provides an efficient HIF library for use in quantitative analyses. Two indices were employed to estimate the potential utility of the HIF library. One of these indices was “coverage on genome”, defined as the probability that any given genomic region will be covered by at least one RH interval in a given population. Coverage on genome was relatively high, with slight differences being observed between chromosomes and populations, and ranged between 94.2% for the B73/BY804 population and 99.3% for the KUI3/B77 population on average; the ZONG3/YU87–1 population was an outlier due to its high inbreeding as a result of selfing for more than eight generations (Additional file 9: Table S3). The other index was the “depth of genome coverage”, which refers to the average number of lines containing the RH interval identified for a given genomic region in a given population. For all nine F_6_ populations, the depth was greater than 5; i.e., more than 5 lines containing a target RH interval were identified in a given population on average (Additional file 9: Table S3). The genetic background of those lines was known based on high-density genotyping and was displayed in the database to help select appropriate lines for QTL fine mapping [23] (http://modem.hzau.edu.cn/maizego/Hif/Chromosome/chromosome.jsp). Importantly, up to 66% and 39% of the RH intervals were shorter than 5 Mb and 2 Mb, respectively, providing an excellent starting point for QTL fine mapping and cloning, given the pure backgrounds and short intervals involved (Fig. 3a). For the narrowing of a QTL region using the present RH lines, 5 RH lines covering the whole or partial QTL region could be identified on average, which allowed the QTL confidence region to be divided into several different, smaller intervals. Thus, using the initial progeny test, the QTL could potentially be validated and delimited to smaller regions, depending on local recombination per se.

To empirically estimate the power and resolution of QTL fine mapping using the HIF library, we collected the confidence intervals of 1191 previously released QTLs affecting 19 agronomic traits from 10 populations [16, 19] (Additional file 2: Data S1). We found that appropriate HIF lines could be identified for more than 94% of the mapped QTLs in ten populations. For a given QTL, 10.6 HIF lines containing heterozygous intervals covering or partially covering the target region were identified on average, which led to an average resolution of up to 1.34 Mb, ranging between 0.86 Mb and 2.72 Mb, using all of the identified RH lines. The QTL mapping resolution depends greatly on the number of different RH intervals covering the target QTL regions (i.e., depth). In the present collection, approximately 67% of QTLs were covered or partially covered by at least 6 RH intervals in a given population, which demonstrates that the present HIF library provides an effective solution for the fine mapping of these identified QTLs with a high resolution (Fig. 3b and Additional file 10: Table S4).

The HIF approach was employed to narrow one QTL region. First, different RH lines were selected to cover or partially cover the QTL region. Second, progeny tests for each selected RH line were used to more precisely determine the interval where the QTL was located, based on a t-test. If the QTL interval was not sufficiently small to specify a manageable number of candidate genes to facilitate transgenic validation, additional markers located within the QTL region allowed the identification of additional recombinants within the QTL region and narrowed the QTL to a smaller interval.

**Fig. 4:**
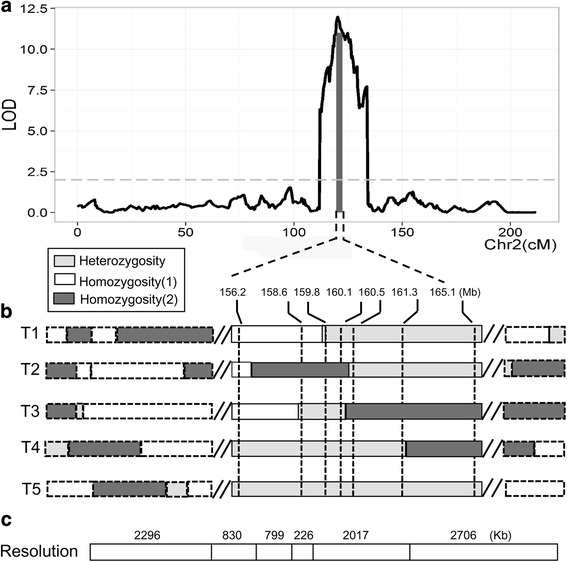
Schematic representation of the fine mapping of a QTL using the HIF approach. (**a**) One major QTL on chromosome 2. The horizontal dashed line indicates the threshold determining whether there a QTL exists. The black shadow indicates the QTL peak and confidence interval. (**b**) HIF types exhibiting heterozygosity within the QTL region. The dashed line indicates the physical boundaries of the heterozygous intervals. The white, black and gray rectangles indicate the identity of two homozygotes and a heterozygote, respectively. (**c**) Potential resolution of QTL refinement based on the one-round HIF approach

To illustrate the power of narrowing QTL regions using the present RH lines (Fig. 4), suppose that a major QTL located on chromosome 2 is identified for the target trait in one RIL population, and the confidence interval is up to 8.4 Mb (Fig. 4a). If 5 different types of residual heterozygous lines are identified, including one line covering the whole QTL region and 4 lines partially covering the QTL region (Fig. 4b), it allows the QTL confidence region to be divided into 6 different intervals ranging from 226 to 2706 Kb. Thus, this hypothetical QTL can potentially be delimited in a single generation to 226 Kb in the best case and 2706 Kb in the worst case, starting from an 8.4 Mb estimate from the initial progeny test (Fig. 4c).

## Discussion

### The genetic basis of RH and its relevance to phenotype

Inbreeding depression is a well-known phenomenon [24], and studies have demonstrated that RH provides a benefit to organisms through higher fitness and disease resistance [6]. In maize, the development of inbred lines is an important process for identifying elite hybrid combinations. However, the properties and biological relevance of RH in advanced inbred lines have rarely been studied. In the maize NAM population, the pericentromeric regions of all ten chromosomes preserve more RH than other regions [6]. However, enrichment of RH in pericentromeric regions was observed on only one chromosome in a maize eight-way MAGIC population [25]. The difference between the two populations may be attributed to statistical bias, in that all lines in NAM share one common parent (B73), whereas the MAGIC lines represent reshuffled genomes from eight parents. In the present collection of 12 advanced inbred populations, we found that only one population (i.e., B73/BY804) exhibited significantly more RH in the pericentromeres, whereas two populations (K22/CI7 and KUI3/B77) showed less RH in the pericentromeres than in the remaining chromosome arms. The remaining nine populations showed no difference in RH rates between the pericentromeres and the remaining chromosome arms (Fig. 1b). These findings suggest that the distribution of RH across chromosomes might be affected by the genetic background. In the B73/BY804 population, three pairs of repulsion-phase linkage QTLs for leaf, tassel branch, ear, and kernel phenotypes have been reported to be located in pericentromeric regions (Additional file 11: Table S5), which provides support for the Hill-Robertson hypothesis explaining the enrichment of RH in pericentromeric regions [6]. Therefore, we conclude that the different patterns of the distribution of RH in the pericentromeres and the remaining chromosome arms (1) are partially in accord with the Hill-Robertson effect, under which linked favorable alleles lead to pseudo-over-dominance and (2) are related to the genetic differences between the two parents.

In the present study, we found that the distribution of RH (including RHR and RHN) was not random across the genome, and 7 specific RH hotspot regions were identified. To inspect the possible bias of RH artifacts due to sequence duplication, we tested whether the identified RH hotspots showed enrichment of paralogous genes based on the B73 reference genome. The results revealed that the number of paralogs at all hotspots, excluding Hot7, was not significantly higher than in random regions across the whole genome (Additional file 4: Figure S3a). Since the genomic sequences vary dramatically between any two maize inbred lines, to some extent, the higher the sequence similarity of paralogs in B73, the more conservative the evaluation of paralogs in non-B73 inbred lines will be. Therefore, we chose paralogs with a similarity higher than 80% to perform the same analysis. The number of paralogous genes at all RH hotspots was not higher than in random regions across the maize genome (*P* > 0.05; Additional file 4: Figure S3b). Thus, we speculated that the excess of paralogs observed at Hot7 may have been caused by ascertainment bias, due to ancient duplications and structural variation between B73 and non-B73 inbred lines, as diminished enrichment of paralogs was observed at Hot7 after stringent filtering of low-similarity genes was implemented. This result was consistent with the findings based on enrichment analysis of repetitive sequences (Additional file 4: Figure S3c). In summary, we understand that sequence duplications, including repetitive sequences and gene paralogs, have the potential to introduce spurious heterozygous genotype calling in the maize genome. However, the analyses based on the experimental data demonstrated that such duplications might be insufficient to induce the RH hotspots observed in the present study unless driven by other forces. Additionally, simulation analysis revealed that the selection responsible for fitness or heterosis might be indispensable for producing the observed RHR pattern in RH hotspots, providing evidence that unknown genetic factors appear to be involved in the determination of RH hotspots. We observed that RHR and RHN were related to the recombination rate, with a stronger effect being observed for RHN than for RHR. It follows that more recombination reshuffles a target interval to produce smaller RH segments. The recombination rate accounted for 0.5% to 13% of the variation of RHR in 12 populations. These estimated values might be affected by parental genome size and structural variations, as the genomic information for all maize lines employed in the present study was based on the B73 reference genome [18, 26]. Thus, our results implied that recombination was an influential but not dominant factor, although it was a major contributor in the NAM population, explaining 35% of the observed variation in heterozygosity rates [27].

To explore the mechanisms underlying RH and its relevance to selection, we evaluated phenotypic performance between heterozygous and homozygous lines at RH hotspots. Several important agronomic traits showed apparent best-parent effects, which might be attributed to the selection of pseudo-over-dominance or over-dominance related to heterosis. To test the pseudo-over-dominance hypothesis, we analyzed the patterns of marker segregation and recombination. We found that the markers within regions on both sides of hotspots either showed no significant distortion in the homozygous parents or were uniformly distorted within a given parent for each RH hotspot. This phenomenon is difficult to explain via the pseudo-over-dominance hypothesis and argues that the excess of heterozygotes resulted from the linkage of two favorable loci in repulsion phase, which can be generated by segregation distortion of alleles towards different parents only on either side of a hotspot. Additionally, many more crossovers were apparent in heterozygotes than in homozygotes across all hotspots, which is congruent with the segregation distortion pattern, indicating that the RH hotspot may not be attributed to selection under the pseudo-over-dominance hypothesis. Therefore, the results implied that over-dominance may be a source of the superiority of excess heterozygosity [3, 4, 28], but the present data do not provide straightforward support for this hypothesis. The reliability of the hypothesis should be further validated based on more experimental evidence in the future. Interestingly, we found that the agronomic phenotypes influenced by the heterozygous region were not relevant from a breeding perspective. For example, tassels produce pollen, and a higher TBN may therefore have been maintained by natural selection for heterozygotes within an RH hotspot, ensuring higher progeny numbers, especially under temperature and moisture stress, by preventing barrenness and poor grain filling. However, an excess TBN would negatively influence yield because of energy consumption for tassel development, and a lower TBN is therefore usually favored in breeding practices [29, 30]. RH hotspots that are important for normal plant growth and development likely establish a balance between plant needs and human needs in agriculturally relevant genetic stocks and may maximize the breeding potential for agricultural traits. Our results give rise to a hypothesis for understanding the biological implications of excess genomic heterozygosity, which has likely avoided elimination due to an advanced history of inbreeding in maize.

To further understand the genetic basis of RH, we identified eight hQTLs regulating RHR variations within the seven RH hotspots, including 3 cis-hQTLs and 5 trans-hQTLs. Combining these results with extensive collected phenotypic data for agronomic traits, we propose a genetic model for the formation of RH hotspots. For the RH hotspots mapped to cis-hQTLs, heterozygotes are probably maintained via balancing selection favoring heterozygotes because of superior plant fitness. Typically, this phenomenon may occur because 1) heterozygotes can compensate for the effects of deleterious mutations on plant fitness, especially for pathogen resistance genes [31]; 2) true over-dominance drives the superiority of heterozygotes over all homozygotes due to antagonistic pleiotropy [32]; and 3) the synergistic effects between two alleles for different phenotypes result in the optimal overall performance and fitness of heterozygotes [33]. However, the high heterozygosity rates at the RH hotspots regulated by trans-hQTLs may not only be selected in the hotspots themselves, but rather, their maintenance may be mediated via selection on the hQTL. Trans-hQTLs apparently function via pleiotropic effects of hQTLs or epistatic interactions between the hotspot and hQTL. Linked gene duplication probably provides an explanation for the nearby trans-hQTL-like hQTL3, and the presence of specific duplications in different populations may explain the observation that hQTLs tend to be population specific. However, the mechanisms underlying the effects of the hQTLs still merit exploration in further studies.

### The library of HIF lines is a rich resource for quantitative genetic studies

The use of RH in RIL populations to construct near-isogenic line populations for QTL fine mapping and cloning has been successfully applied in different species [12–14]. However, this kind of resource is rarely assembled systematically. In the present study, we developed 12 bi-parental linkage populations through single seed descent (SSD), genotyped each family individually and selfed each of them, which provided us the opportunity to build up a large HIF line library for quantitative genetic studies. In total, the HIF library comprises 18,615 unique RH intervals, with an average of 1551 intervals per population, and the total RH intervals in each line extend across approximately 2.8% of the maize genome on average. Approximately 40% of the RH intervals are shorter than 2 Mb, and within each population, an average of more than 4 different lines containing RH intervals can be identified for any given region. We used 1191 QTLs mapped in the 12 populations, affecting 19 agronomic traits, to calculate the real coverage and resolution for QTL fine mapping. The results demonstrated that HIF lines could be identified for 94% of the mapped QTLs, approaching a mapping resolution of 1.34 Mb on average. The high resolution and coverage of the RH intervals make the present collections an ideal resource for QTL fine mapping.

We demonstrated the power of the HIF resource as follows: one hypothetical QTL could potentially be narrowed within one generation to a region of 226 Kb in the best case and 2706 Kb in the worst case, starting from an 8.4 Mb estimate from the initial progeny test. Compared with the traditional backcrossing strategy, the use of the existing HIF family will save a significant amount of time. However, since only 17 diverse inbred lines were employed to develop the RIL populations, the diversity covered by the HIF library might be limited. The genotyping and selfing of each individual when developing an enlarged RIL population or other multiple-parent segregating populations will provide additional resources for the research community.

## Conclusions

A total of 12 advanced inbred populations containing 2196 lines were used to study the patterns of residual heterozygosity across the whole genome of maize. We observed the RH level significantly varied across and along chromosomes and identified seven RH hotspots that excessively enriched heterozygotes in specific regions in the different populations. Simulation analysis suggested that the selection responsible for fitness or heterosis might be indispensable for producing the observed RHR pattern in RH hotspots, and several genetic factors putatively regulate heterozygous rate in RH hotspots. We have identified eight hQTLs (QTL for the heterozygosity rate in RH hotspot regions) significantly affected the RH variations within the RH hotspots, and interpreted the biological meanings and origins of RH intervals in a genetic way in plants, which might open a new insight for genomic evolution study in the future. Furthermore, we showed the HIF library consisting of high-resolution and high-coverage of the RH intervals was a useful resource to the maize community that potentially boost the quantitative genetic studies.

## Additional files

Additional file 1: **Figure S1.** Distribution of RHR in each line in 12 populations and the RH length for all RH intervals. (PDF 179 kb)

Additional file 2: **Data S1.** Summary of QTL intervals for agronomic traits identified in 10 populations. (XLSX 66 kb)

Additional file 3: **Figure S2.** Distribution of residual heterozygosity in the whole genome across 6 populations. (PDF 130 kb)

Additional file 4: **Figure S3.** Enrichment analysis of paralogous genes and repetitive sequences within RH hotspot regions. (PDF 201 kb)

Additional file 5: **Figure S4.** Simulation and enrichment analysis. (PDF 215 kb)

Additional file 6: **Figure S5.** Relationship between residual heterozygosity and recombinant rates. (PDF 294 kb)

Additional file 7: **Table S1.** Allele segregation distortion and recombination in hotspot regions. (XLSX 20 kb)

Additional file 8: **Table S2.** Details of allele segregation distortion in 4 hotspot regions. (XLSX 35 kb)

Additional file 9: **Table S3.** Coverage and depth of the present heterogeneous inbred family line library. (XLSX 20 kb)

Additional file 10: **Table S4.** Empirical resolution for the fine mapping of QTLs using the present HIF library. (XLSX 20 kb)

Additional file 11: **Table S5.** QTLs in repulsion-phase linkage in pericentromeric regions. (XLSX 20 kb)

## Abbreviations

EL: Ear length
EMS: Ethyl methane sulfonate
EW: Ear weight
HIF: Heterogeneous inbred family
HKW: One hundred kernel weight
hQTL: Analysis QTL analysis of heterozygosity rates in RH hotspot regions
KNPR: Kernel number per row of ear
KT: Kernel thickness
KW: Kernel width
LNAE: Leaf number above ear
LW: Leaf width
NAM: Nested association mapping
NIL: The near-isogenic line
QTL: Quantitative trait locus
RH: Residual heterozygosity
RHN: Number of RH intervals
RHR: Residual heterozygosity rate
RIL: Recombinant inbred line
RR: Recombination rate
SSD: Single seed descent
TBN: tassel branch number
TE: Transposon element
ULA: Upper ear leaf angle
