# Supplementary material for: Intraspecific variation of residual heterozygosity and its utility for quantitative genetic studies in maize
Source: BMC Plant Biol. 2018 Apr 19;18:66. doi: 10.1186/s12870-018-1287-4 (PMC5909218; doi:10.1186/s12870-018-1287-4)
Supplement: Supplementary file 1 — Figure S1. Distribution of RHR in each line in 12 populations and the RH length for all RH intervals. (PDF 179 kb) [file 12870_2018_1287_MOESM1_ESM.pdf]

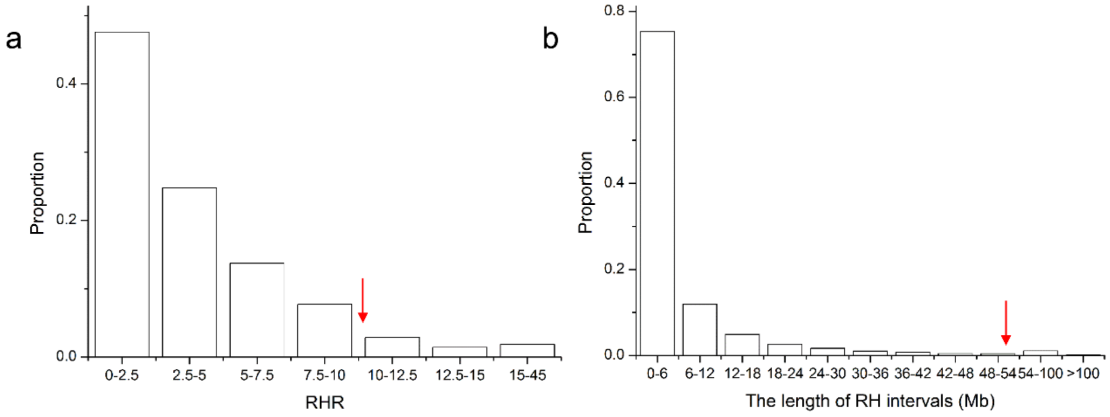

Figure S1. Distribution of RHR in each line in 12 populations and the RH length for all RH intervals. The red arrows mean the RHR threshold is 10% (a) and the length threshold of single RH interval was 50Mb (b).
