# Supplementary material for: Intraspecific variation of residual heterozygosity and its utility for quantitative genetic studies in maize
Source: BMC Plant Biol. 2018 Apr 19;18:66. doi: 10.1186/s12870-018-1287-4 (PMC5909218; doi:10.1186/s12870-018-1287-4)
Supplement: Supplementary file 3 — Figure S2. Distribution of residual heterozygosity in the whole genome across 6 populations. (PDF 130 kb) [file 12870_2018_1287_MOESM3_ESM.pdf]

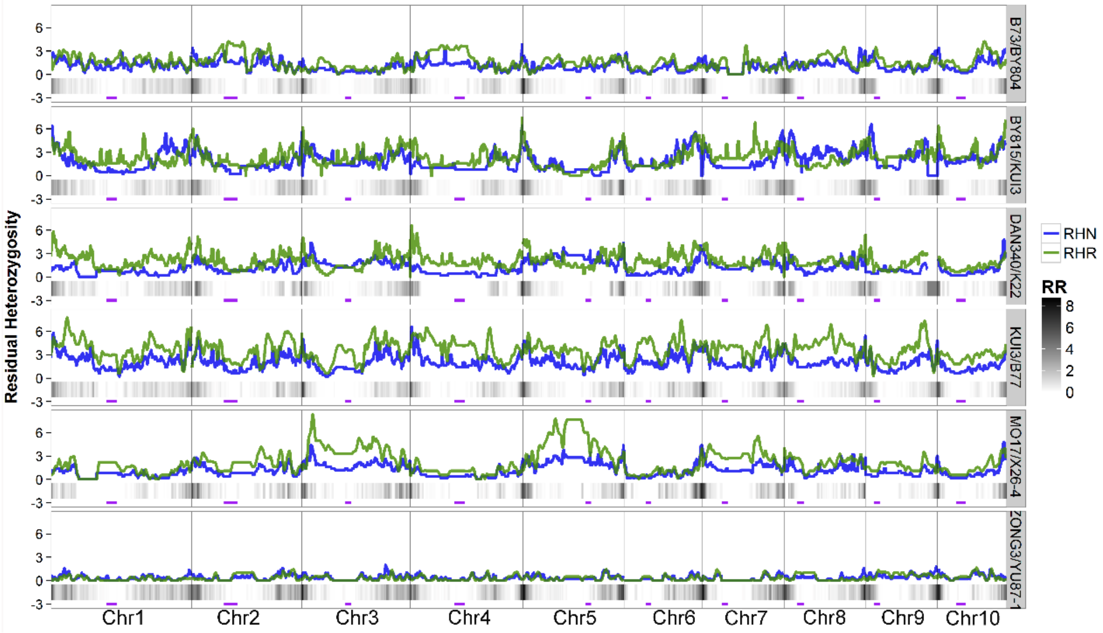

Figure S2. Distribution of residual heterozygosity in the whole genome across 6 populations. The blue lines indicate the number of RH intervals (RHN), and the green lines indicate the RH rate (RHR). The purple rectangles indicate the approximate position of the centromere for each chromosome, and the recombination rate (RR) is shown as a heatmap bar below each population.
