# Supplementary material for: Intraspecific variation of residual heterozygosity and its utility for quantitative genetic studies in maize
Source: BMC Plant Biol. 2018 Apr 19;18:66. doi: 10.1186/s12870-018-1287-4 (PMC5909218; doi:10.1186/s12870-018-1287-4)
Supplement: Supplementary file 4 — Figure S3. Enrichment analysis of paralogous genes and repetitive sequences within RH hotspot regions. (PDF 201 kb) [file 12870_2018_1287_MOESM4_ESM.pdf]

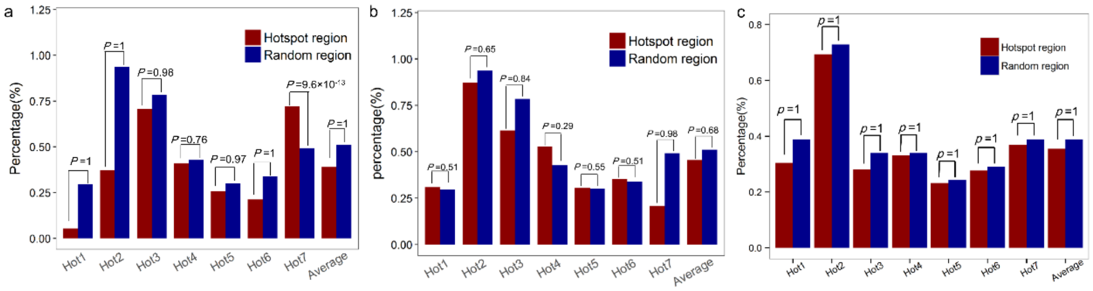

Figure S3. Enrichment analysis of paralogs (ab) and repetitive sequences (c) within RH hotspot regions. (a) The paralogs tagged by polymorphic markers to specifically evaluate the possible bias in identifying RH hotspots per population were chosen. (b) The subset of paralogs in (a) whose similarity were higher than 80% between target and query genes. The binomial distribution was used to test the null hypothesis that both proportions within RH hotspot, the observed paralogs and repetitive sequences proportion (red bar) and the proportion expected by chance (blue bar), come from the same distribution ($P<0.05$; right tail).
