# Supplementary material for: Intraspecific variation of residual heterozygosity and its utility for quantitative genetic studies in maize
Source: BMC Plant Biol. 2018 Apr 19;18:66. doi: 10.1186/s12870-018-1287-4 (PMC5909218; doi:10.1186/s12870-018-1287-4)
Supplement: Supplementary file 5 — Figure S4. Simulation and enrichment analysis. (PDF 215 kb) [file 12870_2018_1287_MOESM5_ESM.pdf]

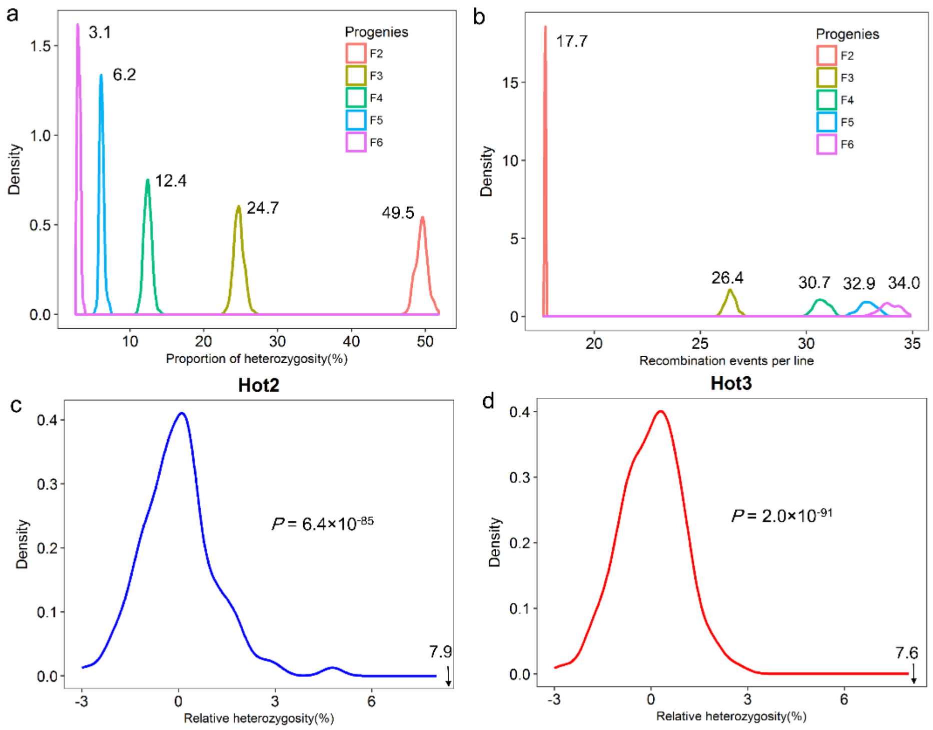

Figure S4. The comparison of distributions for RHR obtained by simulations and observed RHR in Hot2 and Hot3. (a) The simulated distribution of heterozygosity from F_1 to F_6 of 193 lines. (b) The simulated distribution of recombination events per line from F_1 to F_6 of 193 lines. (c-d) *P* means the probability that the observed RHR in Hot2 and Hot3 come from the null distribution via simulation. 7.9% and 7.6% were the observed RHR in Hot2 and Hot3 respectively.
