# Supplementary material for: Intraspecific variation of residual heterozygosity and its utility for quantitative genetic studies in maize
Source: BMC Plant Biol. 2018 Apr 19;18:66. doi: 10.1186/s12870-018-1287-4 (PMC5909218; doi:10.1186/s12870-018-1287-4)
Supplement: Supplementary file 6 — Figure S5. Relationship between residual heterozygosity and recombinant rates. (PDF 294 kb) [file 12870_2018_1287_MOESM6_ESM.pdf]

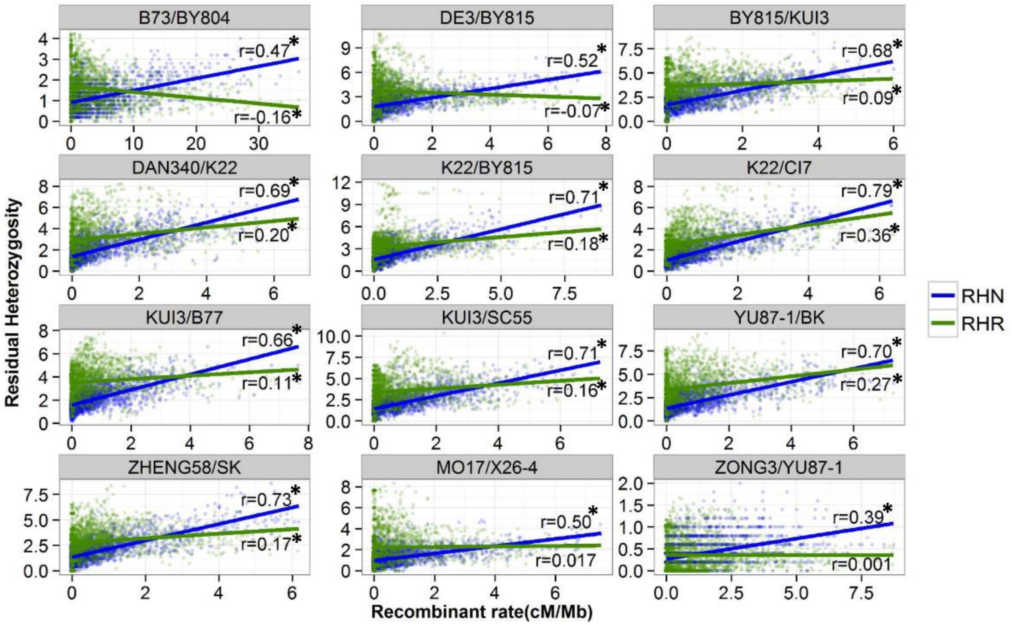

Figure S5. Relationship between residual heterozygosity and recombinant rates. The blue points and lines indicate the correlations of RHN and recombination rate (RR) in 5-Mb window size with 1-Mb walking step, and the correlation coefficient was above the blue lines. The green points and lines represent the correlations of RHR and recombination rate (RR), and the correlation coefficient was below the green lines. The asterisks labelled with correlation coefficient indicate the significance at the level of *P*<0.05.
